# Association Between Remnant Cholesterol and Muscle Mass and Quality: Insights from Muscle Quality Mapping and Abdominal Computed Tomography

**DOI:** 10.3390/diagnostics16111599

**Published:** 2026-05-23

**Authors:** Jung Yoon Moon, Yun Kyung Cho, Eun Hee Kim, Min Jung Lee, Woo Je Lee, Hong-Kyu Kim, Chang Hee Jung

**Affiliations:** 1 Department of Internal Medicine, Asan Medical Center, University of Ulsan College of Medicine, Seoul 05505, Republic of Korea; flytothemm08@gmail.com (J.Y.M.);; 2 Health Screening and Promotion Center, Asan Medical Center, University of Ulsan College of Medicine, Seoul 05505, Republic of Korea

**Keywords:** dyslipidemia, skeletal muscle, lipid metabolism

## Abstract

**Background/Objectives**: Remnant cholesterol (remnant-C), derived from triglyceride-rich lipoproteins, is an important risk factor for cardiometabolic diseases. Given the metabolic link between dyslipidemia and skeletal muscle dysfunction, we aimed to evaluate the association between remnant-C and two key components of sarcopenia—low muscle mass and myosteatosis (ectopic fat deposition in skeletal muscle). **Methods**: This cross-sectional study included 11,570 patients who underwent abdominal computed tomography (CT) for health check-ups. Remnant-C was calculated as total cholesterol minus low-density lipoprotein cholesterol and high-density lipoprotein cholesterol. We conducted multivariable logistic and linear analyses to assess the association between remnant-C and low muscle mass, defined as appendicular skeletal muscle mass divided by body mass index. Additional analysis examined the relationship between remnant-C and myosteatosis, defined using the NAMA (normal attenuation muscle area) divided by TAMA (total abdominal muscle area) index, a novel index derived from muscle quality mapping of abdominal CT scans. **Results:** Low muscle mass was observed in 244 males (3.9%) and 74 females (1.4%). Myosteatosis affected 950 males (15.0%) and 800 females (15.3%). There was an increasing prevalence of both low muscle mass and myosteatosis across remnant-C quartiles. The multivariate-adjusted odds ratios (ORs) for low muscle mass in the highest remnant-C quartile compared with the lowest quartile were 2.17 (95% confidence interval [CI] 1.45–3.26) for males and 1.37 (95% CI 0.68–2.76) for females. The corresponding ORs for myosteatosis were 1.37 (95% CI 1.11–1.69) for males and 1.24 (95% CI 0.96–1.59) for females. **Conclusions**: Elevated remnant-C level is associated with low muscle mass and myosteatosis, especially in male patients. Individuals with higher remnant-C levels may warrant comprehensive evaluation for skeletal muscle health.

## 1. Introduction

Sarcopenia is a progressive skeletal muscle disorder characterized by the loss of muscle mass and function [1]. Muscle function is a critical component in its definition [2,3], as it better predicts adverse health outcomes than muscle mass alone [3,4,5]. In this context, myosteatosis—ectopic fat deposition in skeletal muscle—has gained attention as an indicator of muscle quality and function [6,7,8]. While muscle biopsy remains the gold standard for evaluating myosteatosis, its clinical utility is hindered by its invasive nature [8]. To overcome this limitation, several studies have focused on developing imaging-based indices for myosteatosis [9]. Computed tomography (CT) has been suggested as an effective tool for assessing body fat distribution and overall muscle composition based on tissue attenuation [7,10].

Building upon these advances in CT-based assessment, recent studies have applied artificial intelligence (AI)–driven analytic approaches to generate detailed muscle quality maps [11]. At the L3 vertebral level, the total abdominal muscle area (TAMA) is segmented into inter/intramuscular adipose tissue (IMAT) and skeletal muscle area (SMA). The SMA is further divided into normal attenuation muscle area (NAMA), representing good quality muscle with low fatty infiltration, and low attenuation muscle area (LAMA), reflecting poor quality muscle with high fatty infiltration. The NAMA/TAMA index, calculated by dividing NAMA by TAMA and multiplying by 100, has been proposed as a robust imaging biomarker for evaluating myosteatosis [8,11,12].

The NAMA/TAMA index has been reported to be associated with various cardiometabolic diseases [13,14,15,16,17], especially with dyslipidemia [18]. These findings highlight the crosstalk between lipid metabolism and skeletal muscle. Dyslipidemia may promote ectopic fat distribution to skeletal muscle, manifesting as myosteatosis [19]. Conversely, lipid accumulation in muscle cells can impair insulin signaling, increase mitochondrial oxidative stress, and produce proinflammatory cytokines, thereby contributing to insulin resistance and metabolic dysfunction [19,20].

Despite the established link between myosteatosis and dyslipidemia, comprehensive investigations into this association remain limited. Most existing studies have focused solely on muscle mass [21,22,23,24]—the traditional definition of sarcopenia—or have addressed only a limited range of lipid parameters [18]. Remnant cholesterol (remnant-C), the cholesterol content of remnant lipoprotein particles derived from triglyceride (TG)-rich lipoproteins including very-low-density lipoprotein, intermediate-density lipoprotein, and chylomicron remnants [25], has been insufficiently investigated in relation to muscle health.

Remnant-C is increasingly recognized as a highly atherogenic lipid fraction owing to its permeability to arterial intima, greater cholesterol content per particle, and pro-inflammatory properties [26]. It contributes to residual cardiovascular risk despite LDL-lowering therapy [27] and has been associated with various diseases such as type 2 diabetes, nonalcoholic fatty liver disease, ischemic stroke, and aortic valve stenosis [28,29,30,31].

However, whether remnant-C is associated with alterations in muscle mass and quality, particularly myosteatosis, remains unclear. Therefore, we aimed to identify the relationship between remnant-C and two key components of sarcopenia—muscle mass and myosteatosis—applying the conventional cutoffs for muscle mass and the novel NAMA/TAMA index from abdominal CT analysis and muscle quality mapping.

## 2. Materials and Methods

### 2.1. Study Population

A total of 23,311 individuals aged 20 years or older who underwent abdominal CT scans as part of routine health check-ups at the Health Screening and Promotion Center of Asan Medical Center (Seoul, Republic of Korea) between January 2012 and December 2013 were identified.

Exclusion criteria included the following: individuals on lipid-lowering medications, those with subclinical or overt thyroid dysfunction (thyroid-stimulating hormone [TSH] < 0.4 mIU/L with free thyroxine [T4] > 1.9 ng/dL, or TSH > 5.0 mIU/L with free T4 < 0.8 ng/dL), chronic renal insufficiency (estimated glomerular filtration rate < 60 mL/min/1.73 m^2^), hepatic disorders such as liver cirrhosis, hepatitis B, and hepatitis C, a history of cardiovascular disease or malignancy, and those currently taking glucocorticoids or hormone replacement therapy. Individuals with excessive alcohol intake (>30 g/day in males; >20 g/day in females) were also excluded. Additionally, negative remnant-C values were considered as outliers and excluded.

### 2.2. Biochemical Measurements (Including Remnant-C)

After overnight fasting, early morning blood samples were drawn from the antecubital vein into vacuum tubes and subsequently analyzed at a central certified laboratory in Asan Medical Center. Measurements included concentrations of fasting plasma glucose (FPG), glycated hemoglobin A1c (HbA1c), homeostasis model assessment of insulin resistance (HOMA-IR), high-sensitivity C-reactive protein (hsCRP), liver enzymes, and lipid parameters. All enzyme activities were measured at 37 °C. FPG levels were measured through an enzymatic colorimetric method using a Toshiba 200 FR autoanalyzer (Toshiba Medical System Co., Ltd., Tokyo, Japan). HbA1c levels were obtained using ion-exchange high-performance liquid chromatography (Bio-Rad Laboratories, Inc., Hercules, CA, USA). HOMA-IR was calculated as fasting serum insulin (µIU/mL) × fasting glucose (mg/dL) divided by 405. Furthermore, hsCRP was measured using an immunoturbidimetric method (Toshiba Medical System Co., Ltd., Tokyo, Japan). Fasting total cholesterol (TC), high-density lipoprotein cholesterol (HDL-C), LDL cholesterol (LDL-C), and TG levels were measured through an enzymatic colorimetric method using a Toshiba 200FR Neo (Toshiba Medical System Co., Ltd., Tokyo, Japan). Remnant-C levels were calculated as TC minus LDL-C and HDL-C.

### 2.3. Body Composition Measurements and Definition of Low Muscle Mass

Body compositions, including body fat mass, visceral fat mass, lean body mass, skeletal muscle mass, and appendicular skeletal muscle mass (ASM), were measured through direct segmental multi-frequency bioelectrical impedance analysis (BIA) using the InBody 720 (InBody Co., Ltd., Seoul, Republic of Korea). Low muscle mass was defined as ASM (kg) divided by body mass index (BMI) (kg/m^2^) < 0.789 for males and <0.512 for females, according to The Foundation for the National Institutes of Health Sarcopenia Project [32].

### 2.4. CT Image Collection

Abdomen and pelvis CT scans were conducted using the Somatom Definition scanner (Siemens Healthineers, Erlangen, Germany), Discovery CT750 HD scanner (GE Healthcare, Milwaukee, WI, USA), and LightSpeed VCT scanner (GE Healthcare). All CT scans were performed with the following parameters: 120 kVp; automated dose modulation (CareDose 4D, Siemens Healthineers; automA and smartmA, GE Healthcare); a matrix size of 512 × 512; and collimation of 0.625 mm. Furthermore, image data were reconstructed with a slice thickness of 5 mm using the filtered back-projection technique and a soft tissue reconstruction algorithm (B30f kernel; Siemens Healthineers; Standard kernel, GE Healthcare). For contrast enhancement, 100–150 mL of iopromide (Ultravist 370 or Ultravist 300; Bayer Schering Pharma, Berlin, Germany) was administered intravenously using an automatic power injector.

### 2.5. Assessment of Myosteatosis

Cross-sectional CT images were automatically interpreted using an AI-based program with the segmentation technique of a fully convolutional network [33,34], which was designed to select the inferior endplate level of the L3 vertebra and delineate the boundaries of TAMA, visceral fat area (VFA), and subcutaneous fat area (SFA). TAMA encompassed all muscles visible in the axial image, including the psoas, paraspinal, transversus abdominis, rectus abdominis, quadratus lumborum, and internal and external obliques. An image analyst and a radiologist, blinded to clinical information, reviewed and validated all selected CT images and segmented areas. The AI-based algorithm has been previously validated on an internal and an external dataset using the Dice similarity coefficient and cross-sectional area error [33].

**Figure 1:**
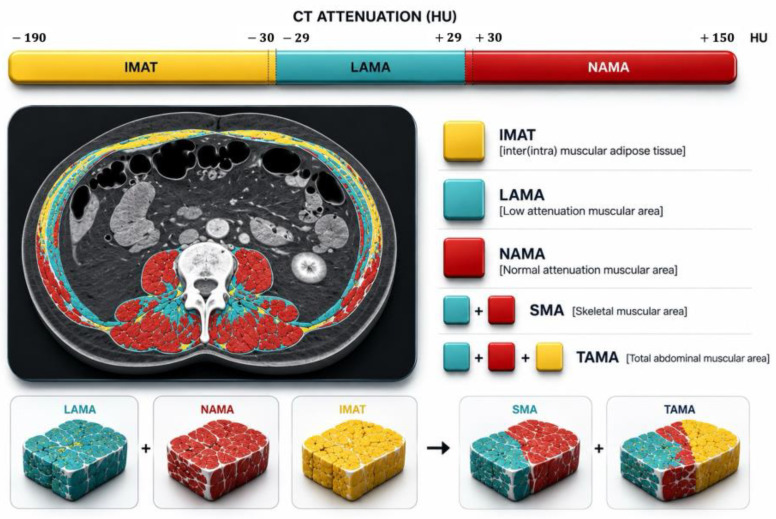
Schematic diagram of abdominal muscle segmentation based on CT attenuation. Modified from Kim et al. [8]. Abbreviations: CT, computed tomography; HU, Hounsfield unit; IMAT, inter/intramuscular adipose tissue; LAMA, low attenuation muscle area; NAMA, normal attenuation muscle area; SMA, skeletal muscle area; TAMA, total abdominal muscle area.

TAMA was further classified based on CT density to evaluate myosteatosis: (1) NAMA (+30 to +150 Hounsfield units [HU]), indicating healthy, nonfatty muscle with minimal intramuscular fat; (2) LAMA (−29 to +29 HU), representing fatty muscles with intramuscular lipid accumulation; and (3) IMAT (−190 to −30 HU), representing visible fat located between muscle groups and fibers [11]. SMA (−29 to +150 HU) included NAMA and LAMA. The NAMA/TAMA ratio was calculated by dividing NAMA by TAMA and multiplying by 100. VFA and SFA were also evaluated based on a specific fat tissue threshold (−190 to −30 HU) (Figure 1).

We defined patients with the NAMA/TAMA index T-score below −1 as having myosteatosis. T-score cutoff was adopted from Kim et al. [11], who established age- and sex-specific reference values for the NAMA/TAMA index in a large Korean health screening population (<73 in men and <72 in women).

### 2.6. Clinical, Lifestyle, and Anthropometric Covariates

We used binary sex categorization designated at birth. All patients completed a questionnaire covering their medical and surgical history, medication use, and health behaviors, including smoking, alcohol consumption, and exercise habits. Smoking status was categorized into three groups: current, past, and never smokers. Alcohol consumption was measured in g/day based on the frequency and amount of drinking, as well as the alcohol content of the beverages consumed. Regular exercise was defined as engaging in moderate-intensity aerobic exercise for at least 30 min, 5 days per week; vigorous-intensity aerobic exercise for at least 20 min, 3 days per week; or resistance exercise at least 3 days per week.

Height and weight were measured while wearing light clothing without shoes. Waist circumference (WC) was measured midway between the costal margin and the iliac crest after a normal expiration. BMI was calculated as weight (kg) divided by the square of height (m). Blood pressure (BP) was checked using an automatic manometer on the right arm after taking at least 5 min of rest.

Hypertension was defined as having a systolic or diastolic BP of 140/90 mmHg or higher or taking antihypertensive medications. Diabetes mellitus was diagnosed if any of the following criteria were met: FPG level ≥ 126 mg/dL (7.0 mmol/L), HbA1c ≥ 6.5%, or current use of antidiabetic medications.

### 2.7. Statistical Analyses

Patients were divided into four groups according to the quartile of remnant-C values for each sex. Statistical analyses were conducted separately for male and female patients to account for differences in muscle mass and attenuation values [7,11,35]. Continuous variables with normal distributions are presented as the mean ± SD, and those with skewed distributions are presented as the median and interquartile range. Categorical variables are shown as counts and percentages. Differences between groups were compared using the one-way analysis of variance method and the Kruskal–Wallis test for continuous variables with post hoc analysis using the Tukey and Dunn method and the Pearson’s chi-squared test for categorical variables. In addition, the Cochran–Armitage test was used to assess for linear trends. Multivariable logistic regression models were used to analyze odds ratios (ORs) and 95% confidence intervals (CIs) for low muscle mass defined by ASM/BMI and myosteatosis defined by the NAMA/TAMA index. In model 1, we adjusted for age, and in model 2, we adjusted for age, VFA/SFA, smoking status, alcohol consumption, and exercise. In fully adjusted model 3, we adjusted for age, VFA/SFA, smoking status, alcohol consumption, exercise, hypertension, and diabetes. For female patients, model 3 was further adjusted for menopause status. Multivariable linear regression analysis was also performed using the same covariates as in the logistic regression models. All analyses were performed using R statistical software (v4.5.2; R Foundation for Statistical Computing, Vienna, Austria). A *p*-value of <0.05 was considered statistically significant.

## 3. Results

### 3.1. Baseline Clinical and Biochemical Characteristics by Remnant-C Level

**Figure 2:**
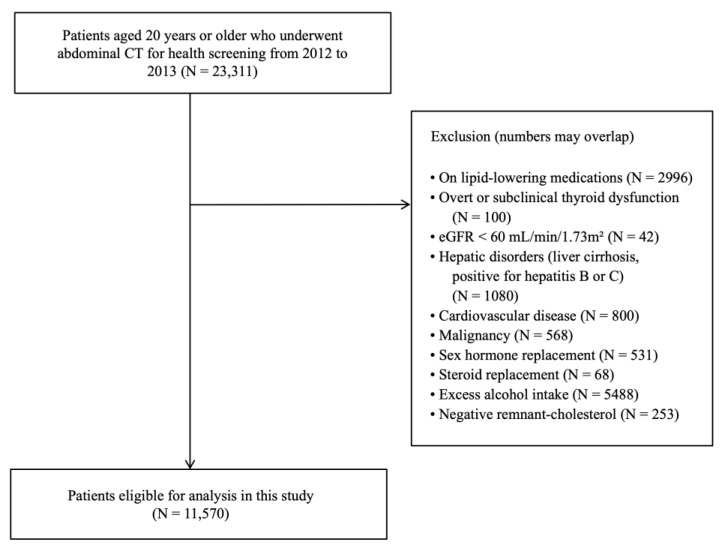
A flow diagram illustrating the selection of the study population. Abbreviations: CT, computed tomography; eGFR, estimated glomerular filtration rate.

A total of 11,570 patients, consisting of 6355 male and 5215 female patients aged between 20 and 87, were included in the final analysis (Figure 2). Baseline characteristics according to remnant-C quartiles are presented separately for each sex (Table 1). In both sexes, individuals in higher remnant-C quartiles showed adverse metabolic profiles, such as higher BMI, WC, BP, FPG, HbA1c, and HOMA-IR levels. They also showed higher TC, LDL-C, TG, and hsCRP levels and lower HDL-C levels. Female patients in the higher remnant-C quartiles tended to be older than those in lower remnant-C quartiles, though this trend was not evident in males. Furthermore, individuals with higher remnant-C levels showed unfavorable health behaviors, including a lower rate of regular physical activity and a higher prevalence of current smokers among males. The prevalence of diabetes and hypertension also increased progressively across higher quartiles.

### 3.2. Body Composition and Myosteatosis Parameters by Remnant-C Level

Body composition and myosteatosis parameters obtained from BIA and CT muscle quality mapping are shown for each sex (Table 2). Body fat mass and visceral fat mass increased progressively from Q1 to Q4, reflecting differences in fat accumulation across quartiles. Lean body mass and skeletal muscle mass showed an upward trend across quartiles, and the ASM/BMI ratio declined progressively from Q1 to Q4, indicating that individuals in higher remnant-C quartiles have a relatively lower proportion of skeletal muscle mass relative to BMI.

Regarding CT-based muscle quality parameters, although absolute NAMA increased across remnant-C quartiles in male patients, both NAMA/BMI and the NAMA/TAMA index declined progressively in both sexes. Conversely, absolute LAMA increased consistently from Q1 to Q4 in both sexes, reflecting greater fatty infiltration of skeletal muscle. The VFA/SFA ratio also showed a consistent increase from Q1 to Q4, indicating greater visceral fat accumulation in higher quartiles. Overall, individuals in higher remnant-C quartiles exhibited greater body fat mass, visceral fat mass, and absolute skeletal muscle mass; however, muscle mass relative to BMI and good muscle quality indicated by the NAMA/TAMA index decreased as remnant-C increased.

### 3.3. Association Between Remnant-C and Low Muscle Mass

**Figure 3:**
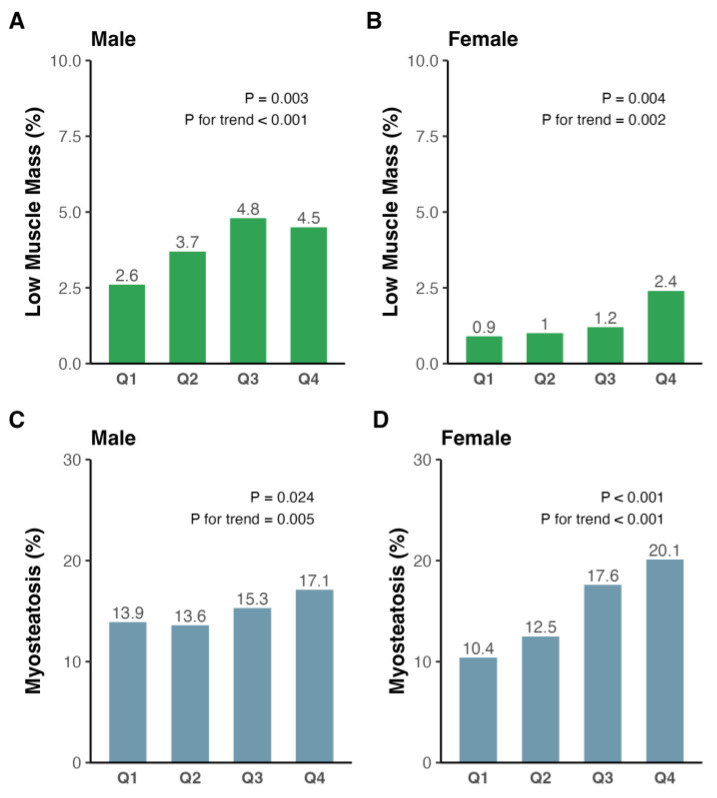
Prevalence of low muscle mass (**A**,**B**) and myosteatosis (**C**,**D**) according to remnant cholesterol quartile.

A total of 244 male (3.9%) and 74 female patients (1.4%) were defined as having low muscle mass using the ASM/BMI criteria. The prevalence of low muscle mass increased progressively across remnant-C quartiles, from Q1 to Q4, both in males (2.6%, 3.7%, 4.8%, and 4.5%; *p* for trend < 0.001) and females (0.9%, 1.0%, 1.2%, and 2.4%; *p* for trend = 0.002) (Figure 3).

**Figure 4:**
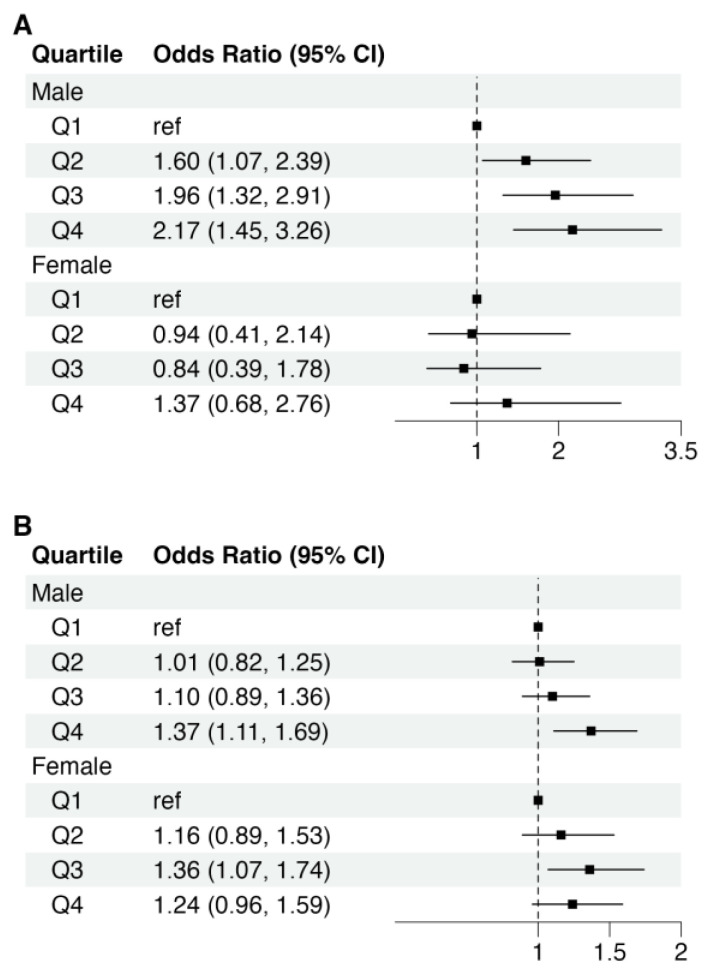
Adjusted odds ratios for low muscle mass and myosteatosis. Forest plot shows adjusted odds ratios in Model 3 for (**A**) low muscle mass and (**B**) myosteatosis across remnant cholesterol quartiles from Q1 to Q4 (Q1 as the reference, indicated by the dashed vertical line).

In male patients, remnant-C quartiles were significantly associated with low muscle mass in all models. In the fully adjusted Model 3, the highest quartile group showed significantly greater odds of low muscle mass compared with those in the lowest quartile group (OR: 2.17, CI: 1.45–3.26). In female patients, the overall association between remnant-C quartiles and low muscle mass remained significant in all models. However, the individual OR for the highest quartile compared with the lowest quartile was not significant in Model 3 (Table 3 and Figure 4).

### 3.4. Association Between Remnant-C and Myosteatosis

A total of 950 males (15.0%) and 800 females (15.3%) were identified as having myosteatosis. The prevalence of myosteatosis increased progressively across remnant-C quartiles, from Q1 to Q4 in both males (13.9%, 13.6%, 15.3%, and 17.1%; *p* for trend = 0.005) and females (10.4%, 12.5%, 17.6%, and 20.1%; *p* for trend < 0.001) (Figure 3).

In the unadjusted model, an increasing trend was observed in ORs across remnant-C quartiles in both males and females. In males, ORs (95% CI) increased from 0.97 (0.80–1.19) in Q2 to 1.11 (0.91–1.35) in Q3 and 1.27 (1.05–1.54) in Q4, compared with Q1 as the reference. In females, the trend was more pronounced, with ORs of 1.23 (0.95–1.58), 1.84 (1.47–2.31), and 2.13 (1.70–2.67) from Q2 to Q4, respectively (Table 4).

In male patients, the increasing trend in ORs across remnant-C quartiles remained significant in all models, with Q4 exhibiting the highest OR. After adjusting for various covariates in Model 3, Q4 presented a 37% increased association of myosteatosis compared with Q1 (OR: 1.37, CI: 1.11–1.69) (Table 4 and Figure 4). Similarly, in female patients, there was a statistically significant association between remnant-C quartiles and the odds of myosteatosis in all regression models. However, unlike male patients, the third quartile group exhibited the highest ORs in Models 2 and 3 (OR: 1.39, CI: 1.09–1.78 and OR: 1.36, CI: 1.07–1.74, respectively).

In multivariable linear regression analysis, each 1 mg/dL increase in remnant-C was independently associated with a decrease in the NAMA/TAMA index in both male (β = −0.029, 95% CI: −0.045 to −0.013) and female patients (β = −0.059, 95% CI: −0.087 to −0.031), after adjustment for age, VFA/SFA, smoking status, alcohol consumption, regular exercise, hypertension, and diabetes (Supplementary Table S1).

## 4. Discussion

In this large-scale cross-sectional study, the prevalence of low muscle mass and myosteatosis increased progressively across remnant-C quartiles. The associations remained significant after adjusting for age, health behaviors, and comorbidities, with some sex-specific variations. These results suggest a potential association that may warrant evaluation for muscle mass and quality among individuals with elevated remnant-C levels.

Individuals with elevated remnant-C levels demonstrated unfavorable metabolic profiles, characterized by increased adiposity (higher BMI, WC, total and visceral fat mass, VFA/SFA), impaired glucose metabolism (higher FPG, Hba1c, and HOMA-IR with a greater prevalence of diabetes), atherogenic dyslipidemia (higher TC, TG, LDL-C and lower HDL-C), and systemic inflammation as indicated by elevated hsCRP levels. These findings are consistent with prior studies linking remnant-C to metabolic disorders such as diabetes and steatohepatitis [28,29], as well as chronic systemic inflammation [36]. Our results suggest that remnant-C may be associated with a broader state of metabolic dysregulation characterized by excess adiposity and adverse body composition.

Both relative muscle mass (ASM/BMI) and muscle quality (NAMA/TAMA index) were significantly lower with increasing remnant-C. After adjusting for potential confounding factors, the overall association between remnant-C quartiles and low muscle mass remained statistically significant across all regression models. This relationship is consistent with previous findings: Jang et al. reported that individuals in the highest remnant-C quartile had significantly increased odds of low muscle mass (OR: 1.33, 95% CI: 1.06–1.68) [23], and Yin et al. demonstrated a positive association between the remnant-C to HDL-C ratio and low muscle mass (OR: 1.55, 95% CI: 1.16–2.07) [24]. Prior studies have also reported associations between myosteatosis and adverse lipid profiles, particularly elevated triglyceride-rich lipoproteins [37,38]; however, the specific role of remnant-C has not been directly investigated. To our knowledge, this is the first study to explore the relationship between remnant-C and myosteatosis, thereby extending previous research that associated a higher NAMA/TAMA index with a lower risk of atherogenic dyslipidemia [18]. The application of the NAMA/TAMA index allowed for quantification of muscle quality, potentially complementing conventional assessments of muscle mass.

In female patients, although a statistically significant overall association for low muscle mass was observed in all models, individual quartile-specific ORs did not reach statistical significance, with confidence intervals crossing 1.0. This is likely attributable to the very low prevalence of low muscle mass among female participants (74 out of 5215; 1.4%), thereby limiting statistical power to detect quartile-level differences. Notably, females in higher remnant-C quartiles tended to be older, whereas males showed the opposite trend. These findings align with the established life-course trajectories of remnant-C: levels peak in early adulthood and decline after midlife in males, whereas in females they rise sharply after menopause and continue to rise with age [39,40]. The early-life elevation in males may be attributed to early visceral adiposity accumulation, lifestyle factors such as alcohol intake, and possibly to survival bias [41,42]. In contrast, the postmenopausal rise in females is likely mediated by estrogen deficiency and reduced hepatic LDL receptor expression [43]. These distinct trajectories suggest that sex-specific patterns of remnant-C should be considered in future prospective studies.

The underlying mechanisms linking remnant-C to low muscle mass and myosteatosis are not fully understood. Hyperlipidemia promotes local adipose tissue inflammation and fat redistribution from subcutaneous fat to visceral fat and skeletal muscles [19]. At the cellular level, accumulated lipid derivatives in muscle cells impair mitochondrial oxidative phosphorylation, hinder beta-oxidation of fatty acids, and increase reactive oxygen species production [20]. Such alterations favor the generation of lipotoxic intermediates such as diacylglycerol and ceramide, which disrupt insulin signaling via protein kinase C activation and Akt/protein kinase B pathway inhibition [44,45,46]. Elevated oxidative stress further contributes to epigenetic modification of DNA of myofibers, leading to protein degradation of muscle fibers and interfering with neural innervation of muscle [20]. These mechanisms may provide a biological basis for the observed association between remnant-C and myosteatosis.

This study has several limitations. First, as a cross-sectional study, we cannot establish a causal relationship. Second, as our cohort was drawn from a single health screening center with several exclusion criteria applied, the study population represents a relatively healthy subset. Compared with Korean nationwide data [47,48,49], our population showed a lower prevalence of diabetes and hypertension and a higher rate of regular physical activity. Although the median remnant-C level (15 mg/dL) was consistent with a nationwide estimate [50], our findings may not be generalizable to other demographic groups. Third, our dataset lacks functional measures such as handgrip strength and gait speed required for sarcopenia diagnosis [2]; therefore, the present study should be understood as examining sarcopenia-related muscle composition outcomes rather than sarcopenia as a clinical entity. Regarding the myosteatosis threshold, the NAMA/TAMA index T-score below −1 was derived from a Korean health screening population and lacks external validation in diverse populations. While its application across multiple studies has demonstrated consistent associations with cardiometabolic conditions [13,14,15,16,17], population-specific cutoffs remain to be established, and future prospective studies are needed to improve generalizability. Fourth, very low prevalence of low muscle mass in female participants (74 out of 5215; 1.4%) likely resulted in insufficient statistical power to detect significant quartile-level differences, and female-specific findings should therefore be considered exploratory. Fifth, remnant-C was calculated as TC minus HDL-C and LDL-C because directly measured remnant-C levels were unavailable. Although previous studies have shown a strong correlation between calculated and directly measured remnant-C [51], potential misclassification of remnant-C levels cannot be excluded [52]. Finally, CT attenuation-based muscle quality assessment may be influenced by technical factors such as scanner differences and contrast enhancement. However, the NAMA/TAMA index—as a ratio derived from the same CT acquisition—may partially mitigate such variability compared with absolute attenuation values.

## 5. Conclusions

Our study showed that Korean adults with elevated remnant-C levels exhibited a significantly higher prevalence and greater odds of low muscle mass and poor muscle quality. Our findings suggest that individuals with elevated remnant-C levels may benefit from further evaluation for muscle mass and muscle composition. Prospective studies are warranted to clarify whether elevated remnant-C plays a causal role in muscle deterioration.

## Figures and Tables

**Table 1:** Baseline clinical and biochemical characteristics according to remnant cholesterol quartile in (A) male and (B) female patients.

(A)					
	Q1 (N = 1795)	Q2 (N = 1534)	Q3 (N = 1460)	Q4 (N = 1566)	*p*-value
Remnant cholesterol, mg/dL					
Range	[0, 12)	[12, 17)	[17, 23)	[23, 191)	
Median (IQR)	9.0 (6.0–11.0)	15.0 (14.0–16.0)	20.0 (19.0–22.0)	29.0 (26.0–36.0)	<0.001
Age, years	54.4 ± 9.8	53.2 ± 9.2 _a_	53.1 ± 9.0 _a_	51.6 ± 8.5	<0.001
BMI, kg/m^2^	23.4 ± 2.7	24.1 ± 2.7	24.7 ± 2.6	25.5 ± 2.8	<0.001
Waist circumference, cm	84.0 ± 7.8	85.9 ± 7.5	87.8 ± 7.3	89.9 ± 7.2	<0.001
SBP, mmHg	122.7 ± 13.1 _a_	123.1 ± 12.8 _ab_	124.1 ± 12.9 _b_	126.2 ± 13.1	<0.001
DBP, mmHg	77.9 ± 9.9 _a_	78.7 ± 9.8 _ab_	79.4 ± 10.0 _b_	81.3 ± 10.6	<0.001
Fasting glucose, mg/dL	96.0 (90.0–103.0) _a_	97.0 (90.0–104.0) _a_	98.0 (91.0–106.0)	99.0 (92.0–108.0)	<0.001
HOMA-IR	0.8 (0.5–1.4)	1.0 (0.6–1.6)	1.2 (0.8–1.8)	1.6 (1.1–2.3)	<0.001
HbA1c, %	5.5 ± 0.6	5.6 ± 0.6	5.7 ± 0.7 _a_	5.8 ± 0.8 _a_	<0.001
Total cholesterol, mg/dL	182.3 ± 30.2	190.1 ± 29.8	199.1 ± 30.5	213.3 ± 34.0	<0.001
HDL-C, mg/dL	57.1 ± 12.7	51.4 ± 11.3	47.6 ± 9.8	42.2 ± 8.7	<0.001
LDL-C, mg/dL	117.2 ± 27.9	123.7 ± 26.8	131.2 ± 27.7	137.8 ± 30.5	<0.001
Triglyceride, mg/dL	77.0 (61.0–96.0)	96.0 (78.0–117.0)	125.0 (103.0–149.0)	188.0 (154.0–241.0)	<0.001
AST, IU/L	25.0 (21.0–30.0) _ab_	25.0 (21.0–30.0) _ac_	25.0 (21.0–31.0) _bc_	26.5 (22.0–33.0)	<0.001
ALT, IU/L	21.0 (16.0–28.0)	22.0 (17.0–31.0)	24.0 (18.0–33.0)	28.0 (21.0–40.0)	<0.001
eGFR, mL/min/1.73 m^2^	93.9 ± 15.4 _ab_	93.3 ± 14.5 _acd_	92.6 ± 15.6 _bce_	92.2 ± 14.7 _de_	0.006
hsCRP, mg/dL	0.04 (0.02–0.09)	0.05 (0.03–0.10)	0.06 (0.03–0.12)	0.08 (0.04–0.16)	<0.001
Smoking Status, N (%)					<0.001
Current smoker	358 (20.0)	437 (28.5)	499 (34.2)	716 (45.8)	
Never-smoker	569 (31.8)	410 (26.7)	370 (25.3)	268 (17.1)	
Past smoker	863 (48.2)	686 (44.7)	591 (40.5)	580 (37.1)	
Alcohol consumption, g/d	6.4 (1.2–12.1)	5.9 (1.1–13.1)	6.0 (1.1–11.6)	7.2 (1.4–14.0)	0.255
Regular exercise, N (%)	1180 (66.0)	954 (62.2)	811 (55.6)	793 (50.8)	<0.001
Diabetes, N (%)	176 (9.8)	163 (10.6)	189 (12.9)	189 (12.1)	0.023
Hypertension, N (%)	555 (30.9)	502 (32.7)	496 (34.0)	581 (37.1)	0.002
(B)					
	Q1 (N = 1271)	Q2 (N = 1150)	Q3 (N = 1484)	Q4 (N = 1310)	*p*-value
Remnant cholesterol, mg/dL					
Range	[0, 9)	[9, 13)	[13, 19)	[19, 106)	
Median (IQR)	6.0 (3.0–7.0)	11.0 (10.0–12.0)	15.0 (14.0–17.0)	23.0 (20.0–27.0)	<0.001
Age, years	50.8 ± 8.9 _a_	51.3 ± 8.7 _a_	53.2 ± 8.4	54.4 ± 8.1	<0.001
BMI, kg/m^2^	21.6 ± 2.7	22.1 ± 2.8	22.8 ± 2.9	23.9 ± 3.1	<0.001
Waist circumference, cm	75.7 ± 7.9	76.8 ± 7.5	79.1 ± 8.1	82.3 ± 8.0	<0.001
SBP, mmHg	113.9 ± 13.8	115.5 ± 13.9	117.7 ± 14.8	121.1 ± 15.5	<0.001
DBP, mmHg	70.7 ± 10.3	71.8 ± 10.3	73.6 ± 10.4	75.5 ± 11.0	<0.001
Fasting glucose, mg/dL	92.0 (87.0–97.0)	92.5 (88.0–99.0)	95.0 (89.0–100.0)	97.0 (91.0–105.0)	<0.001
HOMA-IR	0.7 (0.5–1.2)	0.8 (0.5–1.3)	1.0 (0.6–1.6)	1.4 (0.9–2.0)	<0.001
HbA1c, %	5.4 ± 0.5 _a_	5.5 ± 0.5 _a_	5.6 ± 0.5	5.7 ± 0.7	<0.001
Total cholesterol, mg/dL	186.5 ± 30.2	194.4 ± 30.2	203.9 ± 31.6	218.7 ± 33.8	<0.001
HDL-C, mg/dL	68.8 ± 13.7	64.7 ± 13.8	59.7 ± 12.9	52.2 ± 11.4	<0.001
LDL-C, mg/dL	112.7 ± 28.0	119.0 ± 27.4	129.0 ± 28.5	141.5 ± 30.3	<0.001
Triglyceride, mg/dL	62.0 (50.0–78.5)	70.0 (58.0–88.0)	89.0 (71.0–111.0)	138.0 (106.0–180.0)	<0.001
AST, IU/L	23.0 (19.0–27.0) _ab_	23.0 (19.0–28.0) _ac_	23.0 (19.0–28.0) _bc_	24.0 (20.0–29.0)	<0.001
ALT, IU/L	16.0 (12.0–20.0) _a_	16.0 (13.0–21.0) _a_	17.0 (13.0–23.0)	19.0 (15.0–27.0)	<0.001
eGFR, mL/min/1.73 m^2^	102.9 ± 16.9 _a_	101.5 ± 17.4 _abc_	100.3 ± 17.4 _bd_	100.3 ± 18.5 _cd_	<0.001
hsCRP, mg/dL	0.03 (0.02–0.06) _a_	0.03 (0.02–0.06) _a_	0.04 (0.02–0.08)	0.06 (0.03–0.13)	<0.001
Smoking Status, N (%)					0.084
Current smoker	34 (2.7)	36 (3.1)	40 (2.7)	42 (3.2)	
Never-smoker	1208 (95.1)	1069 (93.0)	1401 (94.6)	1241 (94.9)	
Past smoker	28 (2.2)	44 (3.8)	40 (2.7)	25 (1.9)	
Alcohol consumption, g/d	0.5 (0.0–2.3) _a_	0.4 (0.0–1.9) _ab_	0.4 (0.0–1.7) _b_	0.0 (0.0–1.4)	<0.001
Regular exercise, N (%)	740 (58.3)	659 (57.4)	821 (55.4)	688 (52.7)	0.022
Diabetes, N (%)	54 (4.2)	39 (3.4)	77 (5.2)	136 (10.4)	<0.001
Hypertension, N (%)	189 (14.9)	189 (16.4)	306 (20.6)	388 (29.6)	<0.001
Menopause, N (%)	644 (50.7)	620 (53.9)	948 (63.9)	892 (68.1)	<0.001

Data are presented as mean ± standard deviation or median (IQR) for continuous variables and n (%) for categorical variables. Analysis of variance, the Kruskal–Wallis rank sum test, and Pearson’s chi-squared test were used to calculate the *p*-value. The same subscripts imply statistically insignificant difference values in post hoc analysis. Otherwise, post hoc analysis revealed significant differences between the groups. Square bracket indicates an inclusive boundary (≥); parenthesis indicates an exclusive boundary (<). Abbreviations: ALT, alanine aminotransferase; AST, aspartate aminotransferase; BMI, body mass index; DBP, diastolic blood pressure; eGFR, estimated glomerular filtration rate; HbA1c, glycated hemoglobin; HDL-C, high-density lipoprotein cholesterol; HOMA-IR, homeostasis model assessment of insulin resistance; hsCRP, high-sensitivity C-reactive protein; IQR, interquartile range; LDL-C, low-density lipoprotein cholesterol; SBP, systolic blood pressure.

**Table 2:** Body composition and myosteatosis parameters according to remnant cholesterol quartile in (A) male and (B) female patients.

(A)					
	Q1 (N = 1795)	Q2 (N = 1534)	Q3 (N = 1460)	Q4 (N = 1566)	*p*-value
BIA parameters					
Body fat mass, kg	13.7 ± 5.0	15.1 ± 5.2	16.2 ± 5.1	17.6 ± 5.4	<0.001
Lean body mass, kg	54.4 ± 6.1	55.1 ± 5.9	55.7 ± 6.3	56.9 ± 6.5	<0.001
Skeletal muscle mass, kg	30.5 ± 3.7	30.9 ± 3.6	31.3 ± 3.8	32.1 ± 3.9	<0.001
Appendicular skeletal muscle mass, kg	23.2 ± 2.9	23.5 ± 2.8 _a_	23.7 ± 2.9 _a_	24.2 ± 3.0	<0.001
Visceral fat mass, cm^2^	71.4 ± 29.6	79.8 ± 30.4	86.4 ± 30.6	93.9 ± 33.8	<0.001
ASM/BMI	1.00 ± 0.12	0.98 ± 0.12	0.97 ± 0.11	0.95 ± 0.10	<0.001
CT parameters					
SMA, cm^2^	157.7 ± 21.2	160.7 ± 20.6	162.9 ± 21.6	167.9 ± 21.7	<0.001
SMA/BMI	6.76 ± 0.72 _a_	6.70 ± 0.72 _a_	6.62 ± 0.70 _b_	6.61 ± 0.66 _b_	<0.001
NAMA, cm^2^	127.9 ± 21.1	129.9 ± 20.8 _a_	130.7 ± 21.5 _a_	134.2 ± 21.3	<0.001
NAMA/BMI	5.50 ± 0.91 _a_	5.43 ± 0.88 _a_	5.33 ± 0.86 _b_	5.30 ± 0.82 _b_	<0.001
LAMA, cm^2^	28.0 (21.9–35.7)	28.8 (23.6–36.4)	30.2 (24.2–38.2)	31.8 (25.6–39.7)	<0.001
LAMA/BMI	1.20 (0.97–1.48) _a_	1.21 (1.01–1.47) _ab_	1.23 (1.02–1.52) _bc_	1.26 (1.03–1.54) _c_	<0.001
NAMA/TAMA	80.0 (74.8–84.5)	79.4 (74.3–83.6) _a_	78.7 (73.2–83.2) _ab_	78.4 (73.0–82.9) _b_	<0.001
VFA/SFA	0.92 (0.67–1.21)	0.97 (0.73–1.30)	1.08 (0.80–1.39)	1.15 (0.90–1.47)	<0.001
(B)					
	Q1 (N = 1271)	Q2 (N = 1150)	Q3 (N = 1484)	Q4 (N = 1310)	*p*-value
BIA parameters					
Body fat mass, kg	15.2 ± 5.0	15.8 ± 5.0	17.3 ± 5.4	19.0 ± 5.7	<0.001
Lean body mass, kg	39.8 ± 3.9 _ab_	39.7 ± 4.1 _ac_	39.9 ± 4.0 _bc_	40.4 ± 4.3	0.002
Skeletal muscle mass, kg	21.5 ± 2.3 _ab_	21.5 ± 2.4 _ac_	21.5 ± 2.4 _bc_	21.9 ± 2.6	<0.001
Appendicular skeletal muscle mass, kg	16.1 ± 2.0 _ab_	16.0 ± 2.0 _ac_	16.1 ± 2.0 _bc_	16.3 ± 2.1	0.027
Visceral fat mass, cm^2^	72.8 ± 23.2	74.8 ± 22.1	83.5 ± 24.0	91.1 ± 23.2	<0.001
ASM/BMI	0.75 ± 0.10	0.73 ± 0.10	0.71 ± 0.10	0.69 ± 0.09	<0.001
CT parameters					
SMA, cm^2^	106.0 ± 12.9 _ab_	106.1 ± 12.6 _ac_	107.2 ± 12.8 _bc_	109.5 ± 13.6	<0.001
SMA/BMI	4.94 ± 0.64	4.85 ± 0.60	4.74 ± 0.60	4.62 ± 0.55	<0.001
NAMA, cm^2^	82.6 ± 13.4 _ab_	81.9 ± 13.1 _acd_	81.1 ± 13.7 _ce_	81.3 ± 13.6 _bde_	0.015
NAMA/BMI	3.87 ± 0.77	3.76 ± 0.73	3.61 ± 0.73	3.44 ± 0.66	<0.001
LAMA, cm^2^	21.9 (17.4–27.9)	22.7 (18.0–28.7)	24.4 (19.4–31.0)	26.7 (21.5–33.6)	<0.001
LAMA/BMI	1.02 (0.84–1.24)	1.03 (0.85–1.26)	1.08 (0.90–1.32)	1.12 (0.95–1.37)	<0.001
NAMA/TAMA	76.5 (70.1–81.8)	75.4 (68.7–80.7)	73.7 (66.7–79.1)	71.7 (64.8–77.3)	<0.001
VFA/SFA	0.34 (0.24–0.48)	0.37 (0.27–0.51)	0.44 (0.32–0.61)	0.55 (0.40–0.74)	<0.001

Data are presented as mean ± standard deviation or median (IQR). Analysis of variance and the Kruskal–Wallis rank sum test were used to calculate the *p*-value. The same subscripts imply statistically insignificant difference values in post hoc analysis. Otherwise, post hoc analysis revealed significant differences between the groups. Abbreviations: ASM, appendicular skeletal muscle mass; BMI, body mass index; IQR, interquartile range; LAMA, low attenuation muscle area; NAMA, normal attenuation muscle area; SFA, subcutaneous fat area; SMA, skeletal muscle area; VFA, visceral fat area.

**Table 3:** Prevalence and odds ratios for low muscle mass according to remnant cholesterol quartile in (A) male and (B) female patients.

(A)					
	Q1 (N = 1795)	Q2 (N = 1534)	Q3 (N = 1460)	Q4 (N = 1566)	*p*-value
Prevalence, N (%)	46 (2.6)	57 (3.7)	70 (4.8)	71 (4.5)	0.003
Unadjusted	1.00 (ref)	1.46 (0.98–2.17)	1.91 (1.31–2.79)	1.80 (1.23–2.63)	0.003
Model 1	1.00 (ref)	1.65 (1.11–2.46)	2.20 (1.51–3.23)	2.36 (1.61–3.48)	<0.001
Model 2	1.00 (ref)	1.62 (1.08–2.42)	2.03 (1.37–3.00)	2.20 (1.47–3.30)	<0.001
Model 3	1.00 (ref)	1.60 (1.07–2.39)	1.96 (1.32–2.91)	2.17 (1.45–3.26)	<0.001
(B)					
	Q1 (N = 1271)	Q2 (N = 1150)	Q3 (N = 1484)	Q4 (N = 1310)	*p*-value
Prevalence, N (%)	12 (0.9)	12 (1.0)	18 (1.2)	32 (2.5)	0.004
Unadjusted	1.00 (ref)	1.11 (0.50–2.48)	1.29 (0.62–2.69)	2.64 (1.35–5.15)	0.007
Model 1	1.00 (ref)	1.06 (0.47–2.40)	1.01 (0.48–2.13)	1.96 (0.99–3.86)	<0.001
Model 2	1.00 (ref)	0.97 (0.43–2.19)	0.87 (0.41–1.85)	1.52 (0.76–3.06)	<0.001
Model 3	1.00 (ref)	0.94 (0.41–2.14)	0.84 (0.39–1.78)	1.37 (0.68–2.76)	<0.001

Data are presented as ORs (95% CIs) unless otherwise indicated. Model 1 adjusted for age. Model 2 adjusted for age, VFA/SFA, smoking status, alcohol consumption, and exercise. Model 3 adjusted for age, VFA/SFA, smoking status, alcohol consumption, exercise, hypertension, diabetes, and menopause. Abbreviations: CI, confidence interval; OR, odds ratio; SFA, subcutaneous fat area; VFA, visceral fat area.

**Table 4:** Prevalence and odds ratios for myosteatosis according to remnant cholesterol quartile in (A) male and (B) female patients.

(A)					
	Q1 (N = 1795)	Q2 (N = 1534)	Q3 (N = 1460)	Q4 (N = 1566)	*p*-value
Prevalence, N (%)	250 (13.9)	209 (13.6)	223 (15.3)	268 (17.1)	0.024
Unadjusted	1.00 (ref)	0.97 (0.80–1.19)	1.11 (0.91–1.35)	1.27 (1.05–1.54)	0.028
Model 1	1.00 (ref)	1.09 (0.89–1.34)	1.28 (1.04–1.57)	1.69 (1.39–2.06)	<0.001
Model 2	1.00 (ref)	1.03 (0.84–1.27)	1.12 (0.91–1.38)	1.41 (1.14–1.73)	<0.001
Model 3	1.00 (ref)	1.01 (0.82–1.25)	1.10 (0.89–1.36)	1.37 (1.11–1.69)	<0.001
(B)					
	Q1 (N = 1271)	Q2 (N = 1150)	Q3 (N = 1484)	Q4 (N = 1310)	*p*-value
Prevalence, N (%)	132 (10.4)	144 (12.5)	261 (17.6)	263 (20.1)	<0.001
Unadjusted	1.00 (ref)	1.23 (0.95–1.58)	1.84 (1.47–2.31)	2.13 (1.70–2.67)	<0.001
Model 1	1.00 (ref)	1.22 (0.93–1.59)	1.57 (1.24–1.99)	1.66 (1.31–2.11)	<0.001
Model 2	1.00 (ref)	1.18 (0.90–1.54)	1.39 (1.09–1.78)	1.31 (1.02–1.68)	<0.001
Model 3	1.00 (ref)	1.16 (0.89–1.53)	1.36 (1.07–1.74)	1.24 (0.96–1.59)	<0.001

Data are presented as ORs (95% CIs) unless otherwise indicated. Model 1 adjusted for age. Model 2 adjusted for age, VFA/SFA, smoking status, alcohol consumption, and exercise. Model 3 adjusted for age, VFA/SFA, smoking status, alcohol consumption, exercise, hypertension, diabetes, and menopause. Abbreviations: CI, confidence interval; OR, odds ratio; SFA, subcutaneous fat area; VFA, visceral fat area.

## Data Availability

The raw data supporting the conclusions of this article will be made available by the authors on request.

## Supplementary Materials

The following supporting information can be downloaded at: https://www.mdpi.com/article/10.3390/diagnostics16111599/s1, Table S1: Multivariable linear regression analysis for NAMA/TAMA.

## Abbreviations

The following abbreviations are used in this manuscript:

AI	Artificial Intelligence
ASM	Appendicular Skeletal Muscle Mass
BIA	Bioelectrical Impedance Analysis
BMI	Body Mass Index
BP	Blood Pressure
CI	Confidence Interval
CT	Computed Tomography
FPG	Fasting Plasma Glucose
HbA1c	Glycated Hemoglobin A1c
HDL-C	High-density Lipoprotein Cholesterol
HOMA-IR	Homeostasis Model Assessment of Insulin Resistance
hsCRP	High-sensitivity C-reactive Protein
HU	Hounsfield Units
IMAT	Inter- and Intramuscular Adipose Tissue
LAMA	Low Attenuation Muscle Area
LDL-C	Low-density Lipoprotein Cholesterol
NAMA	Normal Attenuation Muscle Area
OR	Odds Ratio
SFA	Subcutaneous Fat Area
SMA	Skeletal Muscle Area
TAMA	Total Abdominal Muscle Area
TC	Total Cholesterol
TG	Triglyceride
TSH	Thyroid-stimulating Hormone
T4	Thyroxine
VFA	Visceral Fat Area
WC	Waist Circumference
