# Supplementary material for: Association Between Remnant Cholesterol and Muscle Mass and Quality: Insights from Muscle Quality Mapping and Abdominal Computed Tomography
Source: Diagnostics (Basel). 2026 May 23;16(11):1599. doi: 10.3390/diagnostics16111599 (PMC13256455; doi:10.3390/diagnostics16111599)
Supplement: Supplementary file 1 [file diagnostics-16-01599-s001.zip › diagnostics-4227097-supplementary.pdf]

Supplementary Table S1. Multivariable linear regression analysis for NAMA/TAMA

	Male (N = 6334)			Female (N = 5193)		
Variable	β	95% CI	P value	β	95% CI	P value
Remnant cholesterol (per 1 mg/dL)	−0.029	−0.045 to −0.013	<0.001	−0.059	−0.086 to −0.032	<0.001
Age (per 1 year)	−0.261	−0.283 to −0.239	<0.001	−0.422	−0.461 to −0.383	<0.001
VFA/SFA	−2.192	−2.602 to −1.782	<0.001	−6.094	−7.109 to −5.079	<0.001
Smoking status (Current vs. Never)	−0.364	−0.858 to 0.130	0.149	−0.630	−1.961 to 0.701	0.354
Alcohol consumption (g/day)	−0.021	−0.043 to 0.001	0.058	−0.052	−0.126 to 0.022	0.165
Menopause status (Post vs. Pre)	—	—	—	−0.789	−1.420 to −0.158	0.014
No regular exercise	−1.582	−1.956 to −1.208	<0.001	−1.926	−2.375 to −1.477	<0.001
Diabetes	−1.243	−1.819 to −0.667	<0.001	−0.432	−1.412 to 0.548	0.388
Hypertension	−1.430	−1.820 to −1.040	<0.001	−1.574	−2.156 to −0.992	<0.001

R^2 = 0.159 (male), 0.283 (female). Models adjusted for age, VFA/SFA, smoking status, alcohol consumption, regular exercise, hypertension, diabetes, and menopausal status (female only).

Abbreviations: β, standardized regression coefficient; CI, confidence interval; VFA, visceral fat area; SFA, subcutaneous fat area; NAMA, normal attenuation muscle area; TAMA, total abdominal muscle area.
